# Sustainable Nanotechnology Approaches for Rapid Food Contaminant Detection and Future Food Safety Systems

**DOI:** 10.3390/nano16140876

**Published:** 2026-07-16

**Authors:** Huy Loc Nguyen, Hong Minh Xuan Nguyen, Thi Bich Ngoc Nguyen

**Affiliations:** 1 Department of Engineering and Technology, Van Hien University, Ho Chi Minh City 72419, Vietnam; 2 Department of Chemical Engineering and Food Technology, Nong Lam University, Ho Chi Minh City 71308, Vietnam; nmxhong@hcmuaf.edu.vn; 3 Department of Water Management and Hydrological Science, Texas A&M University, 400 Bizzell St., College Station, TX 77843-2117, USA

**Keywords:** sustainable nanotechnology, food contaminant detection, nanosensors, food safety, smart packaging, safe-by-design

## Abstract

Food safety systems are increasingly challenged by globalized supply chains, emerging contaminants, and the need for rapid decision-making before contaminated products reach consumers. Although conventional methods remain essential for confirmatory analysis, their dependence on centralized facilities, specialized personnel, and time-intensive workflows limits their suitability for real-time monitoring. Sustainable nanotechnology offers a promising approach to address these limitations by enabling rapid, sensitive, portable, and resource-efficient contaminant detection. This review critically examines recent advances in nano-enabled platforms for detecting foodborne pathogens, toxins, pesticide residues, heavy metals, allergens, and other food-related contaminants. Emphasis is placed on colorimetric, fluorescent, electrochemical, surface-enhanced Raman scattering, and biosensor-based systems employing sustainable nanomaterials, including biopolymer nanoparticles, carbon-based nanostructures, metal and metal oxide nanoparticles, quantum dots, and hybrid nanocomposites. The roles of green synthesis, low-toxicity materials, reduced solvent use, and safe-by-design strategies are evaluated in relation to environmental sustainability and practical implementation. The integration of nanosensors with smart packaging, portable devices, Internet of Things platforms, artificial intelligence, and data-driven risk assessment is also discussed. Key challenges include matrix interference, reproducibility, sensor stability, scalability, regulatory approval, environmental fate, and consumer acceptance. Continued progress will require validated, scalable, and environmentally responsible technologies capable of reliable operation under real-world food system conditions.

## 1. Introduction

Food safety remains a major global public health and sustainability challenge as food systems become increasingly complex, interconnected, and vulnerable to contamination during production, processing, transportation, storage, and consumption. Foodborne diseases can arise from biological hazards, such as pathogenic bacteria, viruses, parasites, and fungi, as well as chemical contaminants, including pesticide residues, veterinary drug residues, mycotoxins, allergens, heavy metals, and environmental pollutants [1]. The World Health Organization recognizes foodborne disease as a major cause of illness worldwide, with contamination possible at any stage of the food production and supply chain [2]. In the United States, foodborne illness is estimated to cause approximately 48 million illnesses and 3000 deaths each year, illustrating the continued need for more effective prevention, monitoring, and response strategies [3].

The growing globalization of food supply chains has made contaminant detection both more urgent and more challenging. Raw materials and finished products often traverse multiple regions before reaching consumers, increasing the risk of microbial growth, cross-contamination, adulteration, and chemical exposure [4]. At the same time, consumer demand for fresh, minimally processed, and ready-to-eat foods has heightened the need for rapid detection technologies that identify hazards before contaminated products enter the market. Traditional food safety monitoring still relies heavily on laboratory-based methods, including microbial culture, polymerase chain reaction, enzyme-linked immunosorbent assay, chromatography, and mass spectrometry [5]. These methods offer high sensitivity, specificity, and confirmatory value; however, they often require trained personnel, expensive instrumentation, centralized laboratories, and extended analysis times. These limitations reduce their usefulness for real-time decision-making in farms, processing facilities, distribution centers, retail environments, and household-level monitoring.

Rapid contaminant detection technologies are therefore essential for shifting food safety systems from reactive responses to preventive control [6]. Instead of waiting for laboratory confirmation after contamination has already occurred, rapid detection platforms can support earlier intervention, reduce outbreak risks, minimize product recalls, and improve supply chain transparency. In this context, nanotechnology has emerged as a powerful tool for detecting food contaminants because nanomaterials possess unique physicochemical properties, including a high surface-area-to-volume ratio, tunable optical behavior, enhanced electrical conductivity, catalytic activity, strong adsorption capacity, and compatibility with biological recognition elements [7]. These properties enable the development of nanosensors and nanobiosensors with improved sensitivity, faster response, lower detection limits, and greater potential for miniaturization compared with many conventional sensing platforms. Recent reviews have highlighted the increasing use of nanomaterials in food sensing, active packaging, and food safety monitoring applications [8].

Nano-enabled detection systems can be designed through several analytical principles. Colorimetric sensors provide visible signal changes that can be interpreted by the naked eye or by smartphone-based imaging, making them attractive for low-cost, field-deployable screening [9]. Fluorescent sensors offer high sensitivity and multiplexing potential through signal enhancement, quenching, or wavelength shifts [10]. Surface-enhanced Raman scattering platforms provide highly specific molecular fingerprints and ultrasensitive detection for foodborne pathogens more rapidly and accurately through plasmonic nanostructures [11]. In addition, nanomaterial-assisted immunosensors, aptasensors, enzyme-based sensors, and molecularly imprinted sensors have been widely explored for detecting pathogens, toxins, pesticides, heavy metals, and adulterants in complex food matrices.

Sustainability has become an increasingly important consideration in developing food safety technologies. While nanotechnology offers strong analytical advantages, some conventional nanomaterials and synthesis methods may involve toxic reagents, high energy consumption, poor biodegradability, or uncertain environmental impacts [12]. As a result, sustainable nanotechnology emphasizes designing safer, greener, and more responsible nano-enabled systems. This includes using biopolymer-based nanoparticles, carbon-based nanostructures, plant-derived reducing agents, biodegradable supports, aqueous synthesis routes, reduced solvent consumption, and safe-by-design strategies [13]. Green synthesis and low-toxicity material selection can improve the environmental compatibility of nanosensors while supporting broader acceptance in food-related applications. These considerations are especially important because nanomaterials used in food systems may raise concerns about migration, persistence, environmental fate, regulatory approval, and consumer perception. Recent studies on engineered nanomaterials for food safety have highlighted the importance of balancing functional performance with safety, biocompatibility, and sustainability considerations [14,15].

Another important direction is the integration of nano-enabled detection with future food safety systems. Food safety is no longer limited to end-point testing; it increasingly depends on continuous monitoring, traceability, predictive analytics, and data-informed decision-making. Nanotechnology can support this transition by enabling smart packaging, freshness indicators, portable biosensors, wearable or handheld devices, and Internet of Things-connected monitoring systems [16]. When combined with artificial intelligence, machine learning, cloud-based data management, and digital risk assessment, nanosensors may help transform food safety from isolated testing to an intelligent, connected monitoring network [17]. Such systems could support real-time hazard identification, spatial mapping of contamination risks, automated alerts, and improved decision-making across the food supply chain.

Despite rapid progress, several barriers continue to limit the translation of nano-enabled detection technologies from laboratory research to practical food safety applications. Food matrices are chemically and physically complex, and components such as proteins, fats, carbohydrates, pigments, salts, and natural enzymes may interfere with sensor performance [18]. Reported nanosensors show excellent sensitivity in buffer systems but reduced reliability in real food samples [19]. Reproducibility, long-term stability, batch-to-batch consistency, and sensor calibration remain major challenges. In addition, practical deployment requires simple sample preparation, low cost, user-friendly operation, scalability, and validation against standard reference methods. Regulatory uncertainty and limited harmonization of nanomaterial safety assessment further complicate commercialization. The European Food Safety Authority has issued guidance on risk assessment considerations for nanomaterials in the food and feed chain, reflecting the need for structured evaluation of particle-related properties and exposure risks [20].

This review examines recent advances in nano-enabled contaminant detection with an emphasis on sustainability, practical implementation, and future system integration, focusing on major classes of food contaminants, sustainable nanomaterials used in sensing platforms, detection mechanisms, and emerging applications in smart packaging and portable monitoring. It also evaluates key challenges related to matrix effects, validation, safety, environmental impact, scalability, and regulatory acceptance. By connecting analytical performance with sustainability and real-world applicability, this review aims to provide a critical perspective on how nanotechnology can contribute to faster, safer, and more resilient food safety systems.

## 2. Review Methodology

This review was designed to provide a structured and critical synthesis of recent advances in sustainable nanotechnology for rapid food contaminant detection and future food safety systems. The methodology was developed to ensure that the selected literature was relevant to three central themes: nano-enabled detection technologies, sustainability-oriented nanomaterial design, and practical integration into food safety monitoring systems.

### 2.1. Literature Search Strategy

A comprehensive literature search was conducted using major scientific databases, including Web of Science, Scopus, PubMed, ScienceDirect, Google Scholar, etc. The search focused on peer-reviewed journal articles, review papers, book chapters, and selected regulatory or technical reports related to nanotechnology-based food contaminant detection [21]. Priority was given to studies published within the last ten years, with stronger emphasis on literature mainly from 2019 to 2026 to capture recent developments in sustainable nanomaterials, rapid sensing platforms, smart packaging, and digital food safety technologies. Older foundational studies were included when they provided essential background on nanomaterial properties, sensing mechanisms, or established detection principles.

The search terms were developed using combinations of keywords related to food safety, contaminant detection, nanotechnology, and sustainability. Representative search strings included: “*sustainable nanotechnology food safety*,” “*nanotechnology food contaminant detection*,” “*nanosensors for foodborne pathogens*,” “*nanobiosensors food safety*,” “*green synthesized nanoparticles food detection*,” “*rapid detection of food contaminants*,” “*electrochemical nanosensors food contaminants*,” “*colorimetric nan sensors food safety*,” “*fluorescent nanosensors food contaminants*,” “*surface-enhanced Raman scattering food safety*,” “*smart packaging nanosensors*,” “*IoT food safety sensors*,” “*AI food contaminant detection*,” “*safe-by-design nanomaterials*,” and “*nanomaterial risk assessment food systems*.”

Boolean operators were used to refine the search process. For example, terms such as “*nanotechnology*” and “*food contaminant detection*,” “*nanosensor*” and “*pathogen*” and “*food*,” “*green synthesis*” and “*nanoparticles*” and “*food safety*,” and “*smart packaging*” and “*nanomaterials*” and “*contaminant detection*” were applied to identify studies directly aligned with the scope of the review.

### 2.2. Inclusion and Exclusion Criteria

Studies were selected based on their relevance to nano-enabled contaminant detection in food systems. Articles were included when they met one or more of the following criteria: (1) reported the development or application of nanomaterials for detecting foodborne pathogens, toxins, pesticide residues, heavy metals, allergens, veterinary drug residues, adulterants, or freshness-related indicators; (2) discussed sustainable or green synthesis of nanomaterials for sensing applications; (3) evaluated colorimetric, fluorescent, electrochemical, SERS-based, immunosensor, aptasensor, or biosensor platforms; or (4) addressed smart packaging, portable detection, Internet of Things integration, artificial intelligence, or data-driven food safety monitoring.

Studies were excluded if they were unrelated to food safety, focused only on therapeutic or biomedical applications without food relevance, lacked sufficient information on sensing performance, or discussed nanomaterials without connection to contaminant detection or food system monitoring. Articles not available in English were also excluded. Conference abstracts, non-peer-reviewed sources, and duplicated publications were not prioritized unless they provided unique technical or regulatory information that could not be obtained from peer-reviewed sources.

### 2.3. Screening and Selection Process

The initial search generated a broad set of publications covering nanomaterials, sensors, biosensors, food contaminants, and sustainable detection technologies. Titles and abstracts were first screened to remove studies outside the scope of the review. The remaining articles were then assessed through full-text reading to determine their relevance, scientific quality, and contribution to the review objectives.

During full-text screening, attention was given to the type of contaminant detected, nanomaterial composition, sensing mechanism, sample matrix, detection limit, response time, selectivity, validation approach, and potential for real-world application. Studies performed only in buffer solutions were considered useful for mechanistic discussion, but greater weight was given to studies that tested real food matrices or demonstrated practical sample preparation. Review articles were used to identify research trends and supporting background, while original research articles were prioritized for detailed discussion of detection performance and material design.

### 2.4. Data Extraction, Sustainability Assessment, and Practical Relevance

Information extracted from each selected study included the contaminant type, nanomaterial composition, sensing mechanism, analytical performance (e.g., detection limit, response time, selectivity), sample matrix, validation strategy, and practical application. Beyond analytical performance, studies were evaluated for sustainability using criteria such as green synthesis, renewable or biodegradable materials, reduced solvent and energy consumption, material safety, waste generation, recyclability, and potential environmental impacts. Practical relevance was also assessed based on ease of operation, portability, cost, sample preparation requirements, storage stability, compatibility with complex food matrices, and integration with smart packaging, smartphone-based platforms, IoT systems, or automated data analysis. Greater emphasis was placed on studies demonstrating validation in real food matrices and practical applicability.

### 2.5. Critical Analysis and Synthesis

The selected literature was analyzed using a narrative synthesis approach. Rather than only summarizing individual studies, this review compares major nanomaterial classes, detection mechanisms, contaminant targets, and sustainability strategies. Emphasis was placed on identifying common strengths, limitations, and research gaps across the field. For example, many nano-enabled sensors achieve low detection limits under controlled conditions, but few studies demonstrate reproducible performance in real food matrices or under industrially relevant conditions. Similarly, green synthesis is frequently described as sustainable, but life-cycle assessment, long-term safety, and regulatory readiness are often insufficiently addressed.

In this review, sustainability is considered a multidimensional concept rather than a single material attribute. It encompasses the use of green synthesis approaches, low-toxicity and food-compatible materials, renewable or biodegradable resources, reduced solvent and energy consumption, minimized waste generation, recyclability or reusability, limited migration and exposure risks, and consideration of life-cycle and end-of-life impacts. Accordingly, nano-enabled sensing platforms were evaluated not only for their analytical performance but also for their environmental, safety, and practical implications for food-system applications.

The review also evaluates the relationship between nanomaterial properties and sensing performance. Properties such as particle size, surface charge, morphology, optical behavior, conductivity, catalytic activity, porosity, and functional group availability were considered in relation to contaminant recognition, signal amplification, selectivity, and sensor stability. This structure–function perspective was used to clarify how specific nanomaterial features contribute to rapid and sensitive contaminant detection.

### 2.6. Scopes and Limitations of the Review

This review focuses on nanotechnology-enabled approaches for rapid food contaminant detection, with emphasis on sustainability and future food safety system integration. It does not provide a complete quantitative meta-analysis because the reported studies vary widely in contaminant type, detection platform, sample matrix, experimental conditions, and performance metrics. Differences in detection limits, units, food matrices, validation protocols, and sample preparation methods make direct statistical comparison difficult across the literature.

Another limitation is that many studies report proof-of-concept sensor development rather than full validation under commercial food processing conditions. Therefore, this review interprets reported performance data carefully and distinguishes between laboratory feasibility and practical deployment potential. Despite these limitations, the selected literature provides a strong basis for identifying current progress, persistent challenges, and future research priorities in sustainable nano-enabled food contaminant detection.

## 3. Food Contaminants Requiring Rapid Nano-Enabled Detection

Food contaminants encompass a diverse group of biological, chemical, and physical hazards that can enter the food chain at any stage, including agricultural production, processing, packaging, storage, transportation, retail handling, or consumer preparation [22]. Unsafe food containing pathogenic microorganisms or chemical substances can cause more than 200 diseases, ranging from acute gastrointestinal illness to long-term toxicological outcomes, including cancers and developmental disorders [23]. The complexity of modern food systems has heightened the need for detection methods that are not only accurate but also rapid, portable, sensitive, and suitable for real-time or near-real-time monitoring. Conventional laboratory methods remain essential for confirmation, yet their reliance on centralized infrastructure limits their use for immediate decision-making across distributed food supply chains [24]. Nano-enabled detection platforms are increasingly explored because they can amplify optical, electrical, magnetic, catalytic, or molecular recognition signals, enabling detection of contaminants at low concentrations in complex food matrices [25]. This section summarizes major contaminant categories that require rapid detection and highlights the analytical challenges that motivate the use of sustainable nanotechnology-based sensing systems.

### 3.1. Biological Contaminants: Foodborne Pathogens, Viruses, and Spoilage Microorganisms

**Figure 1:**
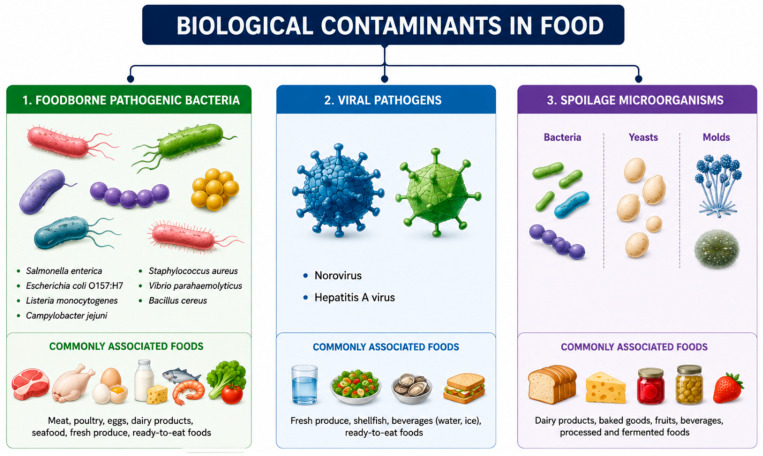
Classification of biological contaminants relevant to food safety (modified by Figure Labs Starter Plan).

Biological contaminants remain among the most urgent targets for rapid food safety detection. As shown in Figure 1, biological contaminants can be broadly classified into pathogenic bacteria, viral pathogens, and spoilage microorganisms.

Pathogenic bacteria such as *Salmonella enterica*, *Escherichia coli* O157:H7, *Listeria monocytogenes*, *Campylobacter jejuni*, *Staphylococcus aureus*, *Vibrio parahaemolyticus*, and *Bacillus cereus* are frequently associated with contaminated meat, poultry, eggs, dairy products, seafood, fresh produce, and ready-to-eat foods [23]. Viral pathogens, including norovirus and hepatitis A virus, are also important because they may persist in foods and require very low infectious doses. In addition, spoilage microorganisms can reduce shelf life, cause economic losses, and produce metabolites that compromise food quality or safety. Because microbial contamination may occur at very low initial levels and then increase during storage or temperature abuse, early detection is critical for preventing contaminated products from reaching consumers [26].

Traditional bacterial detection commonly relies on culture-based enrichment, biochemical identification, immunological assays, or nucleic acid amplification. These methods are reliable but can require several hours to several days, especially when pre-enrichment is needed to recover injured cells or increase pathogen concentration [27]. In contrast, nano-enabled biosensors can improve detection speed through signal amplification and miniaturized recognition interfaces. Gold nanoparticles, magnetic nanoparticles, carbon nanotubes, graphene derivatives, quantum dots, and metal–organic framework-based materials have been incorporated into immunosensors, aptasensors, colorimetric assays, fluorescence sensors, electrochemical sensors, and surface-enhanced Raman scattering platforms [28]. For example, gold nanoparticles can generate visible color changes through aggregation or plasmonic shifts, while magnetic nanoparticles can concentrate bacterial cells from complex samples before detection [29]. Carbon-based nanomaterials and metal nanoparticles can also improve electron transfer in electrochemical pathogen sensors, supporting rapid and sensitive detection using portable devices [30].

Despite these advances, biological contaminant detection remains difficult because food matrices contain fats, proteins, carbohydrates, pigments, enzymes, and naturally occurring microorganisms that may interfere with target recognition or signal generation [31]. Fresh produce, milk, meat homogenates, seafood, and fermented foods present different matrix effects, making universal sensor design challenging. Another limitation is the need to distinguish viable pathogens from dead cells or free DNA, since the presence of genetic material alone does not always indicate an active risk [32]. Future nano-enabled platforms should therefore combine rapid target capture with selective viability assessment, simple sample preparation, and validation in real foods rather than relying only on buffer-based demonstrations.

### 3.2. Natural Toxins and Mycotoxins

Natural toxins are another major group of food contaminants requiring rapid and sensitive detection. Mycotoxins, including aflatoxins, ochratoxin A, fumonisins, zearalenone, patulin, and trichothecenes, are secondary metabolites produced by fungi such as *Aspergillus*, *Penicillium*, and *Fusarium* species [33,34]. These contaminants can occur in cereals, nuts, dried fruits, spices, coffee, milk, and animal-derived products when contaminated feed is consumed. Mycotoxins are especially concerning because many are chemically stable, can persist after processing, and may cause carcinogenic, hepatotoxic, nephrotoxic, immunosuppressive, or endocrine-disrupting effects [35]. Marine, plant, and bacterial toxins also pose food safety concerns, particularly in seafood, grains, and minimally processed foods [36].

Conventional mycotoxin detection often relies on chromatography, mass spectrometry, enzyme-linked immunosorbent assay, or lateral flow immunoassays [37]. While these techniques are valuable, they may require expensive instrumentation, specialized operators, or extraction steps that limit field applicability. Nanotechnology-based detection offers several advantages for toxin monitoring. Plasmonic nanoparticles can support colorimetric assays for aflatoxin and ochratoxin detection; quantum dots and carbon dots can enable fluorescence-based sensing; and SERS-active substrates can provide molecular fingerprinting at trace levels [38,39]. Electrochemical aptasensors have also become attractive because aptamers can provide high specificity toward small-molecule toxins, while nanostructured electrodes improve signal intensity and lower detection limits [40].

A major challenge in toxin detection is that many toxins are small molecules with limited binding sites, making selective recognition more difficult than pathogen detection. Food matrix extraction is also a critical step because toxins may bind strongly to proteins, starches, oils, or other food components. Sustainable nanotechnology may improve this area through greener extraction materials, reusable magnetic sorbents, biodegradable sensor supports, and low-solvent detection workflows [41]. However, practical adoption requires more than low detection limits; sensors must demonstrate accuracy, recovery, repeatability, and stability across representative food matrices and comply with regulatory threshold levels [42].

### 3.3. Chemical Contaminants: Pesticides, Veterinary Drug Residues, Heavy Metals, and Process-Derived Compounds

Chemical contaminants enter food systems through agricultural inputs, environmental pollution, processing conditions, packaging migration, or improper storage. Pesticide residues are common targets for food monitoring because they may remain on fruits, vegetables, grains, and other agricultural products after field application [43]. Veterinary drug residues, including antibiotics, hormones, and antiparasitic agents, may occur in meat, milk, eggs, and aquaculture products if withdrawal periods are not properly followed [44]. Heavy metals such as lead, cadmium, mercury, and inorganic arsenic can accumulate in crops, seafood, rice, and drinking water due to natural geochemical sources, industrial pollution, mining, wastewater irrigation, and contaminated soils [45,46]. Process-derived and persistent environmental contaminants, including acrylamide, furan, polycyclic aromatic hydrocarbons, nitrosamines, chloropropanols, and per- and polyfluoroalkyl substances (PFAS), may form during heating, smoking, fermentation, or refining [47,48]. A sensitive QuEChERS–SPE coupled with an LC-HRMS method was developed for the simultaneous determination of 25 PFAS in diverse food and beverage matrices from the Belgian market [48]. The method achieved excellent sensitivity, with limits of quantification (LOQs) of 0.002–0.3 μg/kg for most analytes. Analysis of 268 commercial samples showed that 43% contained at least one PFAS, with up to 11 PFAS detected in a single wild pork stew sample.

Chemical contaminants present diverse analytical challenges because they differ substantially in molecular structure, polarity, stability, concentration range, and toxicological significance. This heterogeneity limits the applicability of a single detection strategy across different contaminant classes. Although chromatographic and mass spectrometric techniques remain the benchmark for confirmatory analysis due to their high sensitivity and specificity, their dependence on centralized laboratories, sophisticated instrumentation, and extensive sample preparation restricts their use for rapid on-site screening. Nano-enabled sensing platforms have therefore emerged as attractive complementary tools, offering faster analysis, portability, and the potential for field deployment across agricultural production, food processing, retail markets, and border inspection settings. Among these approaches, enzyme-inhibition electrochemical sensors have been extensively investigated for detecting organophosphate and carbamate pesticides, whereas metal nanoparticle-based colorimetric sensors exploit analyte-induced aggregation or catalytic reactions to provide rapid visual detection of selected pesticide residues [49]. For heavy metals, nanostructured electrodes modified with graphene, carbon nanotubes, bismuth films, gold nanoparticles, or metal oxides can improve stripping voltammetry and enable low-level detection of lead, cadmium, mercury, and arsenic [50]. Yang et al. (2023) evaluated ZIF-8 as an adsorbent for removing heavy metal ions from water and assessed the reuse of spent ZIF-8 in cement composites [51]. The results showed that ZIF-8 exhibited strong adsorption performance toward heavy metal contaminants, with adsorption efficiency increasing under favorable pH and contact-time conditions. Kinetic and isotherm analyses indicated that chemisorption and surface complexation played important roles in metal uptake. After adsorption, the waste ZIF-8 was incorporated into cement without significantly compromising mechanical properties. Furthermore, the cement matrix effectively immobilized the adsorbed heavy metals and reduced their leaching potential. These findings demonstrate that ZIF-8 can simultaneously support efficient water purification and sustainable waste reutilization, offering a practical strategy for circular management of MOF-based adsorbents.

**Figure 2:**
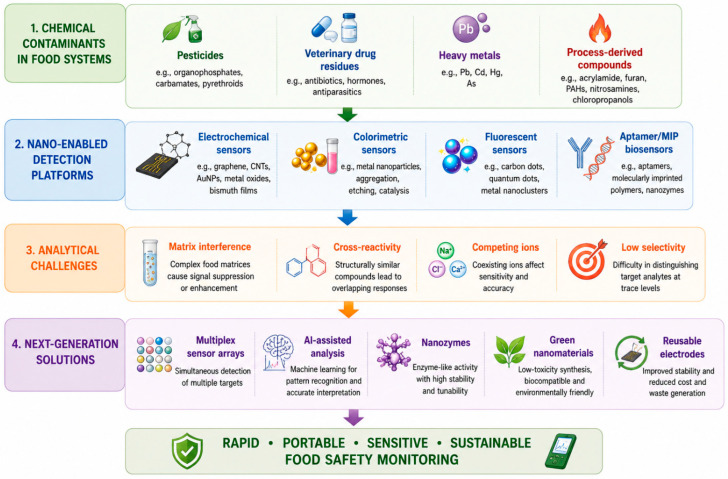
Nano-enabled detection of chemical contaminants in food systems. Original illustration by the authors (modified by Figure Labs Starter Plan).

Fluorescent carbon dots, quantum dots, and metal nanoclusters have also been applied to ion sensing via fluorescence quenching or enhancement [52]. An overview of major chemical contaminants in food systems, nano-enabled detection platforms, analytical challenges, and emerging solutions for rapid and sustainable food safety monitoring is described in Figure 2.

Although chemical nanosensors show strong promise, selectivity remains a central limitation. Structurally similar pesticides or antibiotics may produce overlapping responses, and matrix components can suppress or enhance signals. Heavy metal detection may also be affected by competing ions, pH, salinity, and organic matter [53]. Multiplexed sensor arrays, machine learning-assisted signal interpretation, and hybrid recognition elements such as aptamers, molecularly imprinted polymers, and nanozymes may help address these limitations [54]. From a sustainability perspective, the use of low-toxicity nanomaterials, reusable electrodes, green synthesis, and reduced solvent extraction is essential to ensure that rapid detection does not introduce additional environmental burdens.

### 3.4. Allergens, Adulterants, Packaging Migrants, and Emerging Contaminants

Beyond pathogens and chemical residues, food safety systems must also detect allergens, adulterants, packaging-derived chemicals, microplastics, and other emerging contaminants. Food allergens, such as peanut, milk, egg, soy, wheat, tree nuts, fish, crustacean shellfish, and sesame, can trigger severe immune reactions in sensitive individuals, even at low exposure levels [55]. Adulterants, including undeclared species substitution, melamine, illegal dyes, non-permitted preservatives, and economically motivated substitutions, create both safety and authenticity concerns [56]. Packaging migrants such as bisphenols, phthalates, mineral oil hydrocarbons, and other food-contact chemicals may enter foods during storage, particularly under heat or long contact times [57]. Microplastics and nanoplastics have also received increasing concern due to their widespread occurrence, uncertain toxicological implications, and possible role as carriers for other contaminants [58].

Nano-enabled detection can support this broader contaminant category by improving sensitivity, portability, and multiplexing. Immunosensors and aptasensors can detect allergenic proteins, while SERS and fluorescence platforms can identify adulterants or chemical migrants through spectral or optical signatures [59]. Smart packaging indicators based on nanomaterials may also detect freshness changes, volatile amines, pH shifts, oxygen exposure, or microbial spoilage markers during storage [60]. When combined with smartphones, IoT platforms, and cloud-based analytics, these sensors could support decentralized food safety monitoring across production, logistics, retail, and consumer environments [61].

The main limitation is that many emerging contaminants lack standardized rapid detection protocols, harmonized risk thresholds, or validated reference materials. For allergens, detection must account for processing-induced protein denaturation, cross-contact, and matrix-dependent extraction efficiency. For adulterants and packaging migrants, detection platforms must distinguish intentional fraud from trace background contamination [62]. For microplastics and nanoplastics, particle size, polymer type, additives, and environmental weathering complicate analytical interpretation [63]. Therefore, future nano-enabled systems should be designed not only for high sensitivity but also for regulatory relevance, reproducibility, and compatibility with realistic food safety decision-making.

## 4. Sustainable Nanomaterial Platforms for Rapid Food Contaminant Detection

Sustainable nanotechnology in food contaminant detection is not defined only by analytical sensitivity. A sensor may achieve an extremely low detection limit but still have limited practical value if it requires toxic solvents, unstable reagents, complex fabrication, expensive instrumentation, or materials with uncertain environmental fate. For food safety applications, sustainability requires a broader design framework that considers material origin, synthesis route, toxicity, biodegradability, operational simplicity, waste generation, scalability, and compatibility with real food matrices [64]. Recent literature increasingly emphasizes that nanomaterials used in food sensing and packaging should be evaluated not only for their signal-enhancing properties but also for safety, migration potential, regulatory readiness, and life-cycle impact [65]. EFSA guidance also highlights the need to characterize particle-related properties and assess nanoscale behavior when nanomaterials are used in the food and feed chain, reinforcing the importance of safe-by-design development rather than performance-driven design alone [66].

### 4.1. Green-Synthesized Metallic and Metal Oxide Nanoparticles

Metallic and metal oxide nanoparticles are among the most widely studied nanomaterials for rapid food contaminant detection because of their strong optical, catalytic, electrical, and surface-reactive properties. Gold nanoparticles, silver nanoparticles, copper nanoparticles, zinc oxide, titanium dioxide, iron oxide, cerium oxide, and other metal oxide nanostructures have been applied in colorimetric assays, electrochemical sensors, fluorescence platforms, magnetic separation systems, and SERS-based detection [61,67]. Gold and silver nanoparticles are especially valuable in optical sensing because their localized surface plasmon resonance can produce visible color changes or enhance Raman signals. Iron oxide nanoparticles are useful for magnetic enrichment of pathogens, toxins, or chemical residues from complex food matrices before detection [68,69]. Metal oxide nanoparticles can provide catalytic or semiconducting properties that improve electrochemical and photoelectrochemical sensing responses [70].

However, conventional synthesis of these nanomaterials may involve strong reducing agents, organic solvents, high temperatures, and stabilizers that are not ideal for food-related applications. Green synthesis has therefore become an important strategy for producing metallic and metal oxide nanoparticles using plant extracts, microbial metabolites, polysaccharides, proteins, phenolic compounds, and other bio-derived reducing or capping agents [71]. These biological routes can reduce chemical hazards while introducing functional groups that improve colloidal stability and target interactions. Plant-mediated synthesis is particularly attractive because phytochemicals such as flavonoids, tannins, terpenoids, alkaloids, and organic acids can participate in reduction and stabilization. Microbial synthesis using bacteria, fungi, algae, or yeast may also produce nanoparticles under mild conditions, although process control and downstream purification remain important challenges [72].

In food contaminant detection, green-synthesized nanoparticles can support more sustainable colorimetric and electrochemical platforms for pesticides, heavy metals, mycotoxins, and microbial targets [73]. For example, plasmonic nanoparticles can be designed to aggregate in the presence of specific analytes, producing color changes visible to the naked eye or smartphone camera. Magnetic nanoparticles can reduce sample preparation time by concentrating target cells or molecules from milk, juice, meat extracts, or wash water [74,75]. Metal oxide nanostructures can improve electron transfer, catalytic signal amplification, and surface immobilization of enzymes, antibodies, or aptamers [76]. Despite these benefits, green synthesis should not automatically be treated as safe or sustainable. Batch-to-batch variation in biological extracts, incomplete removal of residual biomolecules, particle instability, and insufficient toxicological evaluation can limit reproducibility and regulatory acceptance [77]. Therefore, future work should report synthesis conditions, particle size distribution, surface chemistry, storage stability, and safety-relevant properties in a standardized manner. Recent reviews also note that green nanoparticles may improve safety and lower toxicity, but their translation still depends on rigorous validation across food matrices and application conditions.

### 4.2. Carbon-Based Nanomaterials and Bio-Derived Carbon Dots

Carbon-based nanomaterials provide another major platform for sustainable food contaminant detection. Graphene, graphene oxide, reduced graphene oxide, carbon nanotubes, carbon nanofibers, carbon black, carbon quantum dots, and biomass-derived carbon dots have been widely used because of their high surface area, chemical tunability, electrical conductivity, fluorescence behavior, and compatibility with miniaturized sensors [60,78]. In electrochemical sensing, graphene and carbon nanotubes can improve charge transfer and increase the effective electrode surface area, allowing more sensitive detection of pesticides, heavy metals, veterinary drug residues, and toxins [79]. In optical sensing, carbon dots and graphene quantum dots can act as fluorescent probes for metal ions, antibiotics, mycotoxins, and freshness indicators through fluorescence quenching, enhancement, or ratiometric signal changes [80].

From a sustainability perspective, carbon dots are especially attractive because they can be synthesized from low-cost and renewable precursors, including fruit peels, plant leaves, starch, cellulose, proteins, amino acids, food waste, and agricultural by-products [81]. This provides an opportunity to convert biological waste streams into functional sensing materials. Biomass-derived carbon dots may also offer lower toxicity than some heavy-metal-containing quantum dots, although toxicity depends strongly on precursor type, synthesis conditions, particle size, surface charge, dopants, and residual impurities [82,83]. Their water dispersibility and photostability make them promising for simple fluorescence assays and paper-based devices. In addition, heteroatom doping with nitrogen, sulfur, phosphorus, or boron can tune fluorescence emission, catalytic activity, and analyte affinity, expanding their use in multiplexed sensing [84].

Graphene-based materials and carbon nanotubes, while powerful for electrical signal amplification, require more careful sustainability assessment. Their production may involve harsh oxidation, strong acids, high energy input, or difficult purification steps. Their persistence and potential biological effects also require attention before food-contact or disposable sensor applications are expanded [85]. In practical food safety systems, carbon nanomaterials may be most suitable when immobilized on stable substrates, electrodes, membranes, or packaging layers to minimize uncontrolled release [86]. Their integration with screen-printed electrodes, flexible sensors, microfluidic devices, and smartphone-based readout systems can reduce sample volume, reagent consumption, and analysis time. The most promising direction is not simply to replace conventional materials with carbon nanomaterials, but to design low-waste platforms that combine renewable precursors, stable immobilization, reusable or recyclable components, and validated performance in representative food matrices.

### 4.3. Biopolymer-Based Nanoparticles, Nanocomposites, and Food-Compatible Supports

Biopolymer-based nanomaterials have become fundamental components of sustainable sensing platforms because they combine renewable origin with functional properties that support environmentally responsible sensor design. In addition to being biodegradable and generally exhibiting low toxicity, many biopolymers possess excellent film-forming ability, chemical versatility, and compatibility with food-contact environments. Chitosan, cellulose nanocrystals, nanocellulose, alginate, starch, pectin, gelatin, whey proteins, zein, silk fibroin, and related natural polymers have therefore been widely explored as nanoparticles, films, hydrogels, membranes, coatings, and immobilization matrices for nano-enabled sensing applications [87]. Beyond serving as passive structural supports, these materials stabilize functional nanomaterials, provide abundant reactive groups for immobilizing biorecognition elements, regulate analyte diffusion, enhance the mechanical integrity of sensing platforms, and help minimize direct exposure to less sustainable or potentially hazardous nanomaterials.

Chitosan is widely used because its amino groups support electrostatic interaction, metal chelation, film formation, and covalent functionalization. In food sensing, chitosan-based composites can immobilize enzymes, antibodies, aptamers, carbon nanomaterials, or metal nanoparticles on electrode surfaces and packaging films [88]. Cellulose nanomaterials are similarly valuable due to their abundance, mechanical strength, high aspect ratio, hydroxyl-rich surface, and ability to form transparent or flexible substrates [89]. Nanocellulose-based paper sensors and films can support low-cost, disposable, and visually readable assays for freshness indicators, heavy metals, pathogens, and chemical residues. Alginate, pectin, gelatin, and protein-based carriers provide additional options for encapsulating dyes, fluorescent probes, or nanoparticles in hydrogel-like matrices that respond to pH, humidity, gases, or microbial metabolites [90,91].

Nanocomposite design allows biopolymers to be combined with inorganic or carbon-based nanomaterials to balance sustainability with analytical performance. For example, a cellulose or chitosan matrix may provide biodegradability and mechanical support, while gold nanoparticles, silver nanoparticles, graphene, or carbon dots provide signal generation [92]. Such hybrid systems are especially relevant for smart packaging, where the sensor must remain stable during storage but respond clearly to spoilage markers, volatile amines, oxygen exposure, or microbial growth. Recent reviews on smart and sustainable food packaging emphasize that nanomaterials can improve barrier properties, mechanical strength, antimicrobial function, and responsiveness to environmental changes [93].

Despite their sustainability advantages, bio-based sensing materials do not inherently guarantee safety, analytical performance, or industrial scalability. The physicochemical properties of biopolymers can vary substantially depending on source material, extraction and purification procedures, molecular weight, degree of deacetylation, crystallinity, and compositional heterogeneity, which may affect swelling behavior, analyte transport, mechanical stability, and sensor reproducibility [94]. Furthermore, food-contact applications require comprehensive evaluation of material migration, potential allergenicity of protein-derived matrices, and interactions with complex food constituents. Future studies should clearly define the functional role of the biopolymer within the sensing architecture rather than treating it solely as a sustainability attribute. Standardized reporting of formulation parameters, film thickness, immobilization strategies, long-term stability, and food-matrix compatibility will be essential to improve reproducibility and facilitate meaningful comparisons across sensing platforms.

### 4.4. Safe-by-Design Principles for Sustainable Nano-Enabled Food Sensors

Safe-by-design development is essential for translating nano-enabled food sensors from laboratory concepts to reliable food safety tools. This approach requires researchers to consider safety, exposure, environmental fate, and end-of-life management during material selection and device design, rather than treating these issues as final-stage concerns. For food contaminant detection, safe-by-design thinking includes choosing low-toxicity materials, reducing hazardous reagents, avoiding unnecessary nanoparticle release, improving sensor stability, minimizing sample and solvent use, and designing devices that can be safely disposed of, recycled, or regenerated [95]. Regulatory guidance for nanomaterials in the food chain also emphasizes physicochemical characterization, particle size distribution, dissolution behavior, exposure assessment, and toxicological evaluation when nanoscale materials are intentionally used in food-related applications [96].

A practical safe-by-design strategy for food sensing materials should integrate hazard-aware material selection, sustainable synthesis, structural containment, and application-specific validation. Material selection should prioritize low-risk and biodegradable alternatives while avoiding nanomaterials associated with persistence, migration, or toxicity concerns. Green synthesis approaches, including aqueous processing and biopolymer- or plant-mediated routes, can further reduce environmental impacts [97]. In addition, immobilization of nanomaterials within films, hydrogels, membranes, paper substrates, and electrode architectures can minimize nanoparticle release while preserving analytical performance [98]. Finally, sensor validation should extend beyond laboratory conditions to include realistic food matrices, storage environments, and benchmarking against established analytical methods to ensure practical applicability and regulatory acceptance.

Sustainability assessments of nano-enabled sensing platforms should extend beyond material selection and manufacturing processes to encompass their operational and end-of-life impacts. Rapid sensing technologies can contribute to more sustainable food systems by reducing sample preparation requirements, minimizing reagent consumption, enabling real-time decision-making, and facilitating the early identification and removal of contaminated products, thereby reducing food waste and resource losses throughout the supply chain [42]. However, the widespread adoption of disposable nanosensors may also introduce additional waste streams and environmental burdens if material recovery, recyclability, and end-of-life management are not adequately considered. Consequently, comprehensive evaluation frameworks incorporating life-cycle assessment (LCA), techno-economic analysis (TEA), and migration studies are needed to compare nano-enabled sensing technologies with conventional analytical methods from both environmental and economic perspectives [99]. Recent advances in intelligent food packaging have further highlighted the importance of integrating sensing functionality with broader sustainability objectives, including food loss reduction, supply-chain traceability, circular material use, and environmental stewardship, rather than focusing exclusively on analytical sensitivity or technological novelty [100].

For the next stage of the field, sustainability should be treated as a measurable design requirement. Authors should report not only the limit of detection and response time, but also the material source, synthesis chemistry, solvent use, stability, reusability, migration behavior, and disposal pathway where relevant. This would allow more meaningful comparisons across nano-enabled platforms and help identify technologies that are both analytically strong and realistic for food-system implementation.

## 5. Nano-Enabled Detection Strategies for Rapid Food Safety Monitoring

Nano-enabled detection platforms translate contaminant–sensor interactions into measurable optical, electrical, catalytic, magnetic, or spectroscopic signals. In contrast to Section 4, which focused on sustainable material platforms, this section examines how these materials are used within sensing architectures to support rapid food safety monitoring. The strongest platforms are not necessarily those with the lowest reported limit of detection, but those that combine sensitivity, selectivity, short response time, simple sample handling, reproducibility, and compatibility with real food matrices. Recent reviews show that nanosensors are increasingly being developed for pathogens, toxins, pesticide residues, heavy metals, allergens, and spoilage indicators, with strong interest in portable, smartphone-readable, and digitally connected formats for on-site screening.

### 5.1. Colorimetric and Fluorescence-Based Nanosensors

A colorimetric nanosensor is among the most practical tools for rapid food contaminant screening because they convert molecular interactions into visible color changes. This makes them attractive for low-resource settings, production-line checks, and preliminary field screening where advanced laboratory instruments are not available [101]. Noble metal nanoparticles, especially gold and silver nanoparticles, are widely used because their localized surface plasmon resonance changes when particle aggregation, dispersion, etching, growth, or surface modification occurs. These changes can be observed visually or quantified using portable imaging tools. Colorimetric nanosensors have been applied to microbial pathogens, mycotoxins, pesticide residues, heavy metals, adulterants, and freshness indicators [102,103]. Their simplicity is a major advantage, but visual interpretation can be subjective when color changes are weak or when food pigments interfere with optical readout.

Fluorescence-based nanosensors provide higher sensitivity and greater quantitative potential than many simple colorimetric assays. Carbon dots, graphene quantum dots, semiconductor quantum dots, metal nanoclusters, upconversion nanoparticles, and fluorescent metal–organic frameworks can respond to contaminants through fluorescence quenching, enhancement, resonance energy transfer, inner-filter effects, or ratiometric signal shifts [104]. Fluorescent platforms are particularly useful for detecting metal ions, antibiotics, mycotoxins, pesticides, and spoilage-related compounds [105]. Carbon dots and biomass-derived fluorescent nanomaterials are increasingly important because they can be synthesized from renewable precursors and may avoid the toxicity concerns associated with cadmium- or lead-based quantum dots [106]. This makes them more compatible with sustainable food safety applications, especially when integrated into paper strips, films, or portable fluorometers.

Despite their considerable analytical advantages, optical nanosensors continue to face important challenges when applied to real food systems. The complexity of food matrices, including inherent color, turbidity, compositional heterogeneity, and high concentrations of proteins, lipids, and other interfering compounds can significantly affect optical signal quality through light scattering, fluorescence quenching, autofluorescence, and nonspecific background responses [107]. As a result, sample pretreatment remains necessary for many food products, particularly dairy products, meat, beverages, spices, edible oils, and fermented foods. Selectivity also remains a critical concern. Certain nanoparticle-based sensing platforms exhibit responses to nonspecific changes in pH, ionic strength, or matrix composition, while fluorescent probes may display cross-reactivity toward structurally related compounds, potentially compromising analytical accuracy [108,109]. To address these limitations, recent sensor designs increasingly integrate nanomaterials with highly selective recognition elements, including antibodies, aptamers, enzymes, and molecularly imprinted polymers, while ratiometric sensing strategies are being adopted to improve signal reliability and minimize matrix-dependent variations [110]. In parallel, smartphone-assisted optical sensing has emerged as a promising approach for portable and low-cost analysis. However, broader implementation will require rigorous standardization of device calibration, illumination conditions, image acquisition protocols, inter-device variability, and data-processing algorithms to ensure reproducibility, comparability, and regulatory acceptance in practical food-monitoring applications [111].

### 5.2. Electrochemical Nanobiosensors and Nanozyme-Amplified Platforms

Electrochemical sensors are highly suitable for rapid food safety monitoring because they are compact, inexpensive, sensitive, and compatible with miniaturized instruments. These platforms convert contaminant recognition into measurable current, potential, impedance, or conductivity changes. Nanomaterials play a central role in enhancing electrochemical sensing performance by increasing the effective electrode surface area, facilitating electron-transfer processes, providing immobilization sites for biorecognition elements, and amplifying catalytic signal transduction. A diverse range of nanomaterials, including graphene and other carbon nanostructures, carbon nanotubes, carbon black, noble-metal nanoparticles, metal oxides, conducting polymers, magnetic nanomaterials, and metal–organic frameworks, have been integrated into electrochemical sensing platforms for the detection of pesticides, veterinary drug residues, heavy metals, foodborne pathogens, allergens, and mycotoxins. Their unique physicochemical properties enable improved sensitivity, lower detection limits, and enhanced analytical performance in complex food matrices [112,113].

Electrochemical biosensors often rely on specific recognition elements such as antibodies, aptamers, enzymes, bacteriophages, lectins, or molecularly imprinted polymers. Aptamer-based sensors are especially attractive for small molecules such as mycotoxins, antibiotics, and pesticides because aptamers can be chemically synthesized, easily modified, and selected against a wide range of targets [114]. Enzyme-based sensors are frequently used for pesticide detection, particularly when organophosphate or carbamate compounds inhibit acetylcholinesterase activity. Immunosensors remain important for pathogens, toxins, and allergens, although antibody stability and cost can limit field use. Molecularly imprinted polymers provide synthetic recognition sites and can improve stability compared with biological receptors, but imprinting quality and nonspecific adsorption remain important concerns [115,116].

**Figure 3:**
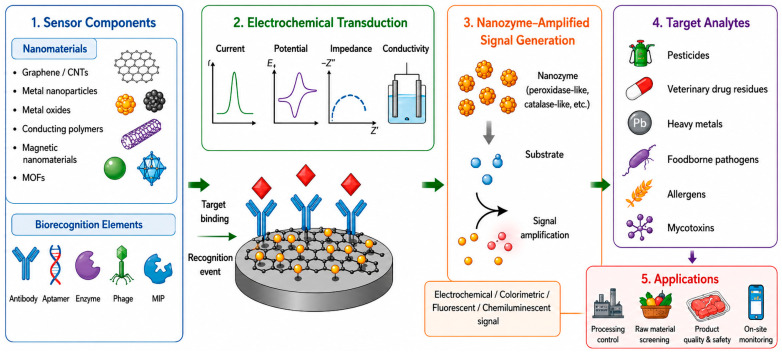
General architecture of electrochemical nanobiosensors and nanozyme-amplified sensing platforms for food contaminant detection (modified by Figure Labs Starter Plan).

Nanozyme-amplified sensing has become a rapidly expanding direction in food contaminant detection. Nanozymes are nanomaterials with enzyme-like catalytic activity, including peroxidase-like, oxidase-like, catalase-like, and superoxide dismutase-like functions. They can replace or supplement natural enzymes in biosensors because they often show better thermal stability, easier storage, lower cost, and tunable catalytic properties [117]. In food safety assays, nanozymes can catalyze colorimetric, fluorescent, chemiluminescent, or electrochemical reactions after target recognition. For example, peroxidase-like nanozymes can promote oxidation of chromogenic substrates, while oxidase-like nanozymes can generate signal changes without added hydrogen peroxide [118]. Recent reviews highlight the growing use of nanozyme-based biosensors to detect food contaminants and the emerging integration of nanozymes with smartphones for portable, on-site analysis. The general design and sensing principle of electrochemical nanobiosensors, including nanozyme-assisted signal amplification, are summarized in Figure 3.

Despite their strong analytical performance, electrochemical and nanozyme-based sensing platforms face several challenges that can affect reproducibility under practical operating conditions. Electrode surfaces are susceptible to fouling by proteins, lipids, salts, and other matrix constituents, often leading to signal drift and reduced long-term stability [119]. Enzyme-inhibition assays provide effective screening capabilities but frequently exhibit responses to multiple target and non-target compounds, limiting their suitability for definitive analyte identification [120]. Similarly, nanozyme catalytic activity can be influenced by environmental and operational factors, including pH, temperature, ionic strength, substrate availability, and surface chemistry [117]. To ensure reliable performance in complex food matrices, sensor development should incorporate appropriate internal standards, antifouling strategies, matrix-specific calibration procedures, and systematic validation against established reference methods [121]. While disposable screen-printed electrodes and paper-based electrochemical devices offer important advantages in terms of portability, affordability, and ease of use, their environmental footprint warrants careful consideration, particularly when fabrication involves metallic nanomaterials, synthetic polymers, or other non-biodegradable components [122].

### 5.3. Surface-Enhanced Raman Scattering and Spectroscopic Nanosensing

Surface-enhanced Raman scattering (SERS) has emerged as one of the most powerful nano-enabled analytical approaches for food safety monitoring due to its ability to generate highly sensitive and chemically specific molecular fingerprints. The technique relies on localized surface plasmon resonance generated by nanostructured substrates, typically composed of gold, silver, or bimetallic nanoparticles, which dramatically enhance Raman scattering signals from molecules located in close proximity to the nanomaterial surface [123]. This signal amplification enables the detection of contaminants at trace or even ultra-trace concentrations, including pesticides, mycotoxins, veterinary drug residues, illegal dyes, adulterants, food additives, marine biotoxins, and pathogen-associated biomarkers. In contrast to many conventional optical sensing methods, SERS provides rich structural information through characteristic vibrational spectra, facilitating both compound identification and multiplex analysis within complex samples. Furthermore, the inherently weak Raman scattering of water minimizes background interference in aqueous systems, making SERS particularly attractive for the analysis of liquid food matrices and food extracts [124].

SERS sensing strategies can generally be categorized as either label-free or label-based. In label-free SERS, the Raman spectrum of the target analyte is directly acquired when the molecule is adsorbed onto or positioned near the plasmonic substrate, enabling intrinsic molecular fingerprinting without additional labeling steps [125]. Although this approach offers analytical simplicity, signal intensity is highly dependent on analyte adsorption behavior, molecular orientation, and proximity to electromagnetic hot spots. In contrast, label-based SERS employs Raman reporter molecules in combination with recognition elements such as antibodies, aptamers, or encoded nanoprobes to generate amplified and more reproducible signals. These platforms are particularly advantageous for sensitive pathogen detection and multiplex analysis, albeit at the expense of increased probe-design complexity [126]. Recent advances have focused on the development of multifunctional SERS architectures, including bimetallic Au–Ag nanostructures, magnetic-plasmonic hybrid probes, and portable Raman instrumentation, further expanding the potential of SERS for rapid and on-site food contaminant monitoring [127].

Despite its exceptional sensitivity, the practical application of SERS remains constrained by reproducibility challenges. Signal enhancement is highly dependent on the formation and distribution of nanoscale electromagnetic hot spots, substrate uniformity, nanoparticle aggregation behavior, excitation wavelength, analyte localization, and adsorption efficiency [125]. Consequently, even minor variations in substrate fabrication or sample preparation can lead to substantial signal fluctuations between measurements. Matrix complexity presents an additional challenge, as food constituents may compete for adsorption sites, hinder analyte access to the plasmonic surface, or generate overlapping spectral signatures that complicate data interpretation [123]. To improve analytical robustness, recent efforts have focused on engineering more reproducible SERS substrates, integrating magnetic separation and preconcentration strategies, incorporating microfluidic sample-processing systems, and employing internal standards for signal normalization [128]. Advances in chemometrics and machine learning have further enhanced the interpretation of complex Raman datasets, enabling improved discrimination of multiple contaminants from noisy or highly overlapping spectra [129]. Nevertheless, although these developments are expanding the practical utility of SERS, the technique is best regarded as a highly informative screening and confirmatory tool that complements, rather than replaces, established reference methods such as chromatography and mass spectrometry.

Beyond the major sensing platforms discussed above, several emerging spectroscopic and optoelectronic approaches are expanding the toolbox available for rapid food safety monitoring. Nanomaterial-enhanced infrared spectroscopy, localized surface plasmon resonance (LSPR) sensors, fluorescence lifetime-based sensing systems, and photoelectrochemical detection platforms offer complementary analytical capabilities for the identification of chemical and biological contaminants [130]. The integration of these technologies with portable instrumentation, fiber-optic sensing architectures, and microfluidic devices is particularly attractive for on-site and real-time analysis. Nevertheless, broader adoption will require continued efforts to reduce instrumentation costs, streamline sample preparation workflows, and develop robust calibration and validation models capable of maintaining analytical accuracy across diverse food matrices and operating conditions [131].

### 5.4. Paper-Based, Lateral Flow, Microfluidic, and Smartphone-Integrated Platforms

A major goal in food contaminant detection is to move testing closer to the point of need. Paper-based devices, lateral flow assays, microfluidic chips, and smartphone-integrated sensors are therefore important because they can reduce assay time, reagent volume, instrument dependence, and operator complexity. Nanomaterials improve these platforms by providing visible labels, fluorescence signals, magnetic enrichment, catalytic amplification, or conductive pathways [132]. Gold nanoparticles remain common in lateral flow strips because they produce stable red-colored bands, while fluorescent nanoparticles, magnetic beads, carbon dots, and nanozymes can improve sensitivity and quantitative readout [133].

Lateral flow assays (LFAs) and aptamer-based strip sensors remain among the most widely adopted platforms for rapid screening of food contaminants, including mycotoxins, allergens, veterinary drug residues, foodborne pathogens, and pesticide residues [41]. Their popularity stems from their low cost, portability, ease of operation, and ability to generate results within minutes, making them particularly attractive for field-based and point-of-need applications. Nevertheless, conventional LFAs often exhibit limited analytical sensitivity and may be susceptible to false-positive or false-negative results arising from matrix interference, nonspecific binding, or variability in signal interpretation [134]. To overcome these limitations, recent studies have incorporated advanced nanomaterial-based signal amplification strategies, including nanozyme labels, fluorescent nanoprobes, SERS nanotags, and smartphone-assisted image analysis, thereby improving sensitivity, quantification capability, and overall assay reliability [135]. Closely related developments in paper-based microfluidic analytical devices (μPADs) have further expanded the functionality of portable sensing platforms [136]. By exploiting capillary-driven fluid transport through patterned paper channels, these systems can integrate multiple analytical operations, including sample preparation, reagent delivery, target recognition, washing, and signal generation within a compact, low-cost, and equipment-free format suitable for decentralized food safety monitoring.

Microfluidic platforms offer stronger control over mixing, separation, incubation, and detection than simple paper strips. They are useful for pathogen capture, nucleic acid amplification, immunoassays, and multiplexed contaminant detection [137]. When combined with magnetic nanoparticles or nanostructured electrodes, microfluidics can support rapid concentration and detection of low-abundance targets. Nevertheless, fabrication cost, clogging, food matrix compatibility, and user operation remain challenges. For routine food safety applications, microfluidic devices must be robust enough for non-laboratory conditions and should not require complex preprocessing that offsets their portability benefits.

Smartphone-integrated nanosensing platforms have emerged as a promising approach for decentralized food safety monitoring by leveraging the multifunctional capabilities of modern mobile devices. In addition to serving as image acquisition tools, smartphones can function as analytical processors, data-transmission platforms, and user interfaces, enabling the quantification of colorimetric, fluorescent, and other optical sensing responses in real time [138]. Integration with cloud-based databases and digital traceability systems further enhances their potential for large-scale food quality and safety surveillance. Recent studies have highlighted the growing role of smartphone-coupled nanozyme and nanobiosensor technologies in delivering rapid, user-friendly, and on-site analytical solutions while facilitating automated data processing and result interpretation [139]. Calabretta et al. (2023) developed a genipin-based colorimetric paper sensor for monitoring food spoilage through the detection of biogenic amines (BAs), which accumulate during microbial degradation of meat products [140]. The sensor produced a visible blue color upon reacting with amines such as putrescine, allowing rapid naked-eye assessment of food freshness. Quantitative analysis using smartphone image processing achieved a detection limit of approximately 0.1 mM putrescine. When integrated into chicken meat packaging, the sensor successfully tracked spoilage under different storage temperatures, with color intensity increasing as freshness declined.

Despite these advantages, analytical reliability remains a critical consideration. Variations in ambient lighting, camera specifications, device models, image-processing settings, data compression, and user operation can significantly influence measurement accuracy and reproducibility. Consequently, broader implementation will require standardized calibration procedures, internal reference systems, controlled imaging environments, and transparent data-processing algorithms to ensure consistent performance and support regulatory and industrial acceptance [141].

Taken together, these portable platforms show how nano-enabled detection can move beyond laboratory proof-of-concept toward practical food safety screening. Their greatest value may lie in tiered monitoring systems: rapid on-site nanosensors can identify suspicious samples, while confirmatory laboratory methods can verify results when legal or regulatory action is required. This approach can improve response speed without compromising analytical reliability.

## 6. Integration of Nano-Enabled Detection into Future Food Safety Systems

The long-term impact of sustainable nanotechnology on food safety will depend not only on advances in sensor performance but also on the successful integration of nano-enabled detection platforms into comprehensive food monitoring and management systems. While laboratory studies frequently emphasize analytical metrics such as sensitivity, detection limits, and response times, effective food safety management requires a broader consideration of reliability, robustness, decision-making value, and operational feasibility [142]. In practice, sensing technologies must function within a larger framework that encompasses representative sampling, data interpretation, traceability, regulatory requirements, risk communication, corrective actions, and sustainability objectives [142]. Consequently, nano-enabled sensors should not be viewed solely as stand-alone analytical devices but rather as components of an integrated preventive food safety infrastructure that combines rapid screening, digital surveillance, risk assessment, and confirmatory analysis. This perspective aligns closely with established food safety management systems such as Hazard Analysis and Critical Control Point (HACCP), which rely on systematic hazard identification, critical control point monitoring, verification procedures, documentation, and corrective actions [143]. When appropriately validated and strategically implemented throughout the food supply chain, nanosensors have the potential to strengthen these functions by enabling more frequent, rapid, and decentralized monitoring. Furthermore, their integration with digital traceability and recall systems may enhance the ability of food producers and regulators to detect hazards, support timely interventions, and improve responsiveness to food safety incidents, thereby contributing to more resilient and sustainable food control systems.

### 6.1. Smart Packaging and Real-Time Monitoring Platforms

**Figure 4:**
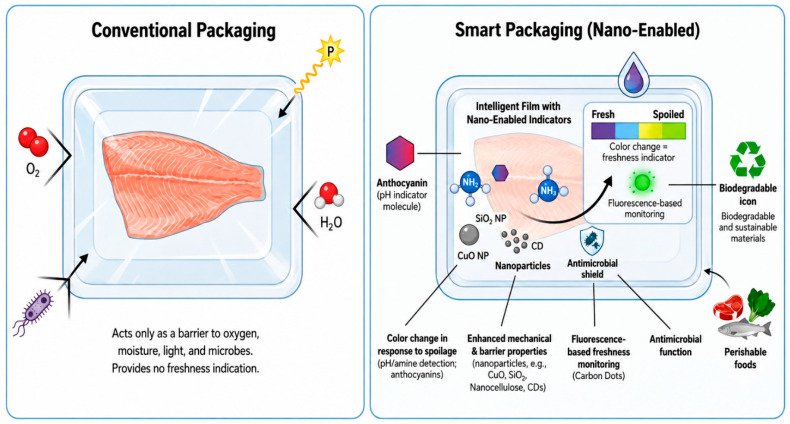
Comparison of Conventional Packaging and Nano-Enabled Smart Packaging Incorporating Freshness Indicators, Antimicrobial Nanomaterials, and Sustainable Components (modified by Figure Labs Starter Plan).

Smart packaging is one of the most practical routes for translating nano-enabled detection from laboratory assays into real food safety systems. Unlike conventional packaging, which mainly acts as a passive barrier against oxygen, moisture, light, and microbial contamination, smart packaging is designed to provide information about the condition of the food during storage, transport, retail display, and consumer handling [144]. In nano-enabled smart packaging, nanomaterials can function as optical indicators, fluorescence probes, antimicrobial components, gas-sensitive elements, or signal-enhancing matrices. These systems are especially relevant for perishable foods, including fish, shrimp, meat, poultry, dairy products, and fresh produce, where spoilage may occur rapidly and where real-time quality indicators can help reduce foodborne risk and unnecessary food waste [145,146]. Figure 4 summarizes the functional differences between conventional and nano-enabled smart packaging, highlighting the incorporation of intelligent sensing elements and antimicrobial nanomaterials to improve food safety, quality monitoring, and shelf-life extension.

A common strategy is the incorporation of pH-sensitive compounds into biopolymer films to monitor spoilage-related metabolites. During the deterioration of fish, shrimp, and meat, microbial and enzymatic reactions generate volatile nitrogenous compounds, including ammonia, trimethylamine, dimethylamine, and other total volatile basic nitrogen compounds [147]. These compounds increase the local pH and can trigger visible color changes in intelligent films. For example, anthocyanin-based indicators have been widely incorporated into chitosan, starch, gelatin, cellulose, and polyvinyl alcohol films because anthocyanins change color in response to pH variation. Published studies have shown that plant-derived anthocyanins from sources such as red cabbage, butterfly pea flower, roselle, purple sweet potato, and black rice can be used to fabricate freshness indicators for fish, shrimp, pork, and chicken products [148,149]. In a recent study, Wu et al. [150] developed anthocyanin-based indicator films modified with SiO_2_ nanoadditives for shrimp freshness monitoring; the films exhibited visible color transitions during refrigerated storage, demonstrating how inorganic nanoadditives can improve sensitivity and visual discrimination in biopolymer-based intelligent packaging.

Chitosan-based intelligent films provide another strong example of sustainable smart packaging. Chitosan is attractive because it is biodegradable, film-forming, antimicrobial, and rich in amino groups that can interact with natural dyes or nanomaterials [151]. Recent reviews have summarized the use of chitosan films containing anthocyanins, betalains, curcumin, alizarin, and other plant-derived indicators for monitoring freshness biomarkers in seafood and meat products [152]. For example, chitosan films containing plant extracts have been reported to respond to volatile amines released during fish spoilage, while also improving mechanical and barrier properties [153]. These systems illustrate how smart packaging can combine sustainability with practical food quality monitoring. However, color stability remains a major limitation because natural pigments can degrade under light, oxygen, heat, and changes in humidity. Another research of Piryaei and Khiavi (2026) developed a starch–chitosan film containing green-synthesized CuO nanoparticles and eggplant peel anthocyanins, which exhibited improved physicochemical, antioxidant, antimicrobial, and intelligent packaging properties [154]. The optimized formulation (85% starch, 10% chitosan, 3% CuO nanoparticles, and 2% anthocyanins) produced a uniform film with a thickness of approximately 15.3 μm and low water vapor permeability (1.013 × 10^−7^ g^−1^ s^−1^ m^−1^ Pa^−1^). Incorporation of CuO nanoparticles increased tensile strength from 11.24 to about 15.5 MPa, while anthocyanins enhanced opacity, antioxidant activity, and pH-responsive color changes.

Therefore, nanostructured carriers, silica nanoparticles, cellulose nanocrystals, clay nanoparticles, and carbon dots have been explored to improve pigment stabilization, optical response, and film performance. Carbon dots have recently received considerable attention as smart packaging components because they offer fluorescence response, pH sensitivity, low toxicity, photostability, and compatibility with biodegradable polymers [155]. Carbon dots can be synthesized from biomass, food waste, fruit peels, amino acids, or plant-derived precursors, making them attractive for sustainable packaging applications.

Mirzajani et al. [156] reported that carbon quantum dot (CQD)-based nano-packaging significantly outperformed essential oil nanocapsules (EO-NCs) in preserving fresh-cut kale during 40 days of refrigerated storage. CQD treatment resulted in the lowest weight loss (5.50 g), compared with EO-NCs (11.92–12.49 g) and the control (27.29 g). CQDs also better retained chlorophyll (12.61 mg g^−1^ FW), ascorbic acid (50.33 mg g^−1^ FW), phenolic compounds (251.97 µg mL^−1^), and antioxidant activity (39.64% DPPH scavenging). Moreover, microbial counts were reduced to 130 CFU mL^−1^, compared with 946 CFU mL^−1^ in untreated samples. The treatment effectively suppressed oxidative enzymes, including catalase and polyphenol oxidase, thereby reducing browning and quality deterioration. Mohammed and Mohammed (2026) reviewed recent advances in carbon dot (CD)-integrated edible films for sustainable food packaging applications [157]. The authors highlighted that incorporating CDs into protein-, polysaccharide-, and lipid-based films significantly improves mechanical strength, UV-blocking capacity, moisture and gas barrier properties, antioxidant activity, and antimicrobial performance. CDs also enable intelligent packaging functions through fluorescence-based monitoring of food freshness, pH changes, and spoilage biomarkers. They emphasized that biomass-derived and heteroatom-doped CDs offer enhanced bioactivity and sensing capabilities. Despite their promising multifunctionality, challenges related to large-scale production, standardization, migration behavior, toxicological assessment, and regulatory approval remain key barriers to commercial implementation.

In biodegradable packaging, carbon dots can also contribute antioxidant, antimicrobial, and barrier-related functions, depending on their synthesis route and surface chemistry [158]. These examples show that smart packaging is moving beyond simple color labels toward multifunctional films capable of sensing, preserving, and communicating food quality changes.

Smart packaging can also be designed to detect gases and environmental changes. Oxygen indicators, carbon dioxide indicators, humidity sensors, and time–temperature indicators can help identify package leakage, cold-chain failure, and storage abuse. Nanomaterials such as TiO_2_, ZnO, silver nanoparticles, graphene oxide, and nanocellulose have been incorporated into packaging films to improve barrier properties, antimicrobial activity, optical responsiveness, and mechanical strength [159,160]. For example, nanocellulose-based films can provide mechanical reinforcement and serve as transparent substrates for colorimetric indicators, while metal oxide nanoparticles can support oxygen- or gas-sensitive responses [161]. These platforms are useful because food safety is determined not only by the presence of contaminants at a single time point; it is also affected by temperature fluctuations, packaging integrity, microbial growth, and gas composition during distribution.

Despite these advances, smart packaging must meet strict practical and safety requirements before commercial adoption. First, the sensing element must be stable throughout the intended shelf life and should not produce false responses caused by harmless food variation. Second, the color or fluorescence change should be easy to interpret by producers, retailers, or consumers. Third, the indicator must not migrate into food at unsafe levels. Fourth, the sensor should be compatible with packaging production methods such as casting, coating, extrusion, electrospinning, printing, or lamination. Recent reviews on bio-based smart packaging emphasize that scalability, cost, regulatory approval, consumer acceptance, and migration behavior remain major barriers to commercialization [162,163]. Therefore, future studies should report not only color response or sensitivity, but also migration testing, film thickness, mechanical strength, water vapor permeability, storage stability, cytotoxicity, where relevant, and performance in real foods under realistic cold-chain conditions.

### 6.2. Digital Connectivity, IoT, and AI-Assisted Food Safety Surveillance

**Figure 5:**
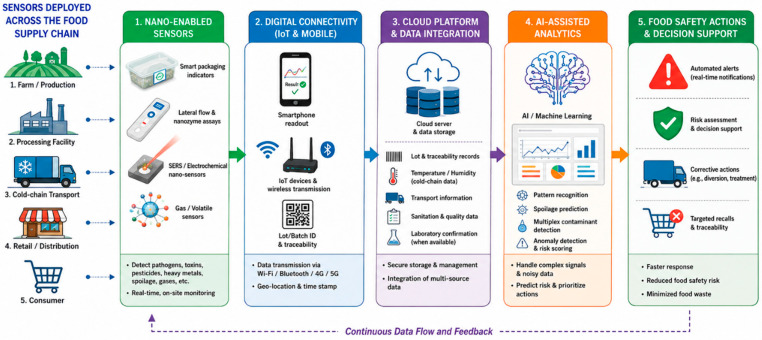
Integration of nano-enabled sensing, digital connectivity, and AI-assisted analytics for real-time food safety surveillance (modified by Figure Labs Starter Plan).

Nano-enabled sensors become more valuable when they are connected to digital food safety systems. A single sensor reading can provide useful information, but a networked sensor platform can support continuous monitoring, trend analysis, automated alerts, and risk-based decision-making. IoT systems can connect sensors embedded in packaging, cold-chain vehicles, storage rooms, processing facilities, and retail environments [164]. These devices can collect data on temperature, humidity, gas composition, spoilage indicators, contamination signals, and package integrity. When sensor outputs are transmitted to cloud-based dashboards, they can be linked with lot codes, supplier records, sanitation data, transport history, and recall systems. This structure allows food safety management to move from isolated testing toward integrated surveillance [165]. Figure 5 illustrates how nano-enabled sensors can be integrated with smartphone-based platforms, IoT networks, cloud-based data systems, and AI analytics to enable real-time food safety surveillance across the supply chain. This connected framework supports continuous monitoring, automated data processing, risk-based decision-making, and targeted interventions.

Within this framework, smartphone-assisted sensing represents one of the most practical and accessible approaches for translating nano-enabled detection technologies into decentralized food safety applications. Smartphone-based sensing is a practical example of digital connectivity in food safety. Smartphones can serve as cameras, optical readers, processors, geolocation tools, and communication devices [166]. Colorimetric nanosensors, fluorescent strips, lateral flow assays, and nanozyme-based assays can be coupled with smartphone imaging to quantify signals that would otherwise be interpreted visually. Qin et al. (2025) reviewed smartphone-based biosensors for food testing and highlighted their use in portable detection of contaminants through optical, electrochemical, and dual-mode readout formats [167]. Similarly, Gbonyea et al. reviewed smartphone-integrated nanozyme technologies for rapid on-site food safety analysis, noting that nanozymes can improve signal amplification while smartphones enable real-time image processing and accessible readout [168]. These systems are valuable because they reduce dependence on centralized laboratories and allow non-expert users to perform preliminary screening.

Specific examples include smartphone-readable colorimetric assays for pesticide residues, heavy metals, mycotoxins, and bacterial contamination. In nanozyme-based pesticide detection, peroxidase-like or oxidase-like nanomaterials can catalyze visible reactions after target interaction, and the resulting color intensity can be analyzed using smartphone applications [169]. This approach has been explored for organophosphate and carbamate pesticide screening because these compounds are difficult to monitor rapidly outside laboratories. Paper-based microfluidic devices combined with portable imaging systems have also been developed for pesticide detection in fruits and vegetables. Researchers reported a compact chemiluminescence-based microfluidic paper analytical device for on-site pesticide detection in food washing water, illustrating how low-cost portable detection can be linked with imaging-based quantification and field use [170]. Although such platforms may not replace chromatography–mass spectrometry, they can serve as rapid screening tools for markets, farms, and processing lines.

Artificial intelligence can further improve nano-enabled detection by interpreting complex signals. Food matrices often generate noisy, overlapping, or nonlinear responses. Machine learning models can help analyze fluorescence spectra, electrochemical curves, Raman spectra, electronic nose data, and colorimetric images. For example, Liu et al. reported a SERS-based approach for simultaneous detection of food contaminants that combined bacterial capture with machine learning, showing how spectral data and computational classification can improve multiplex detection in complex matrices [171]. Machine learning-assisted SERS has also been applied to pesticide detection, where algorithms can classify spectral patterns that are difficult to distinguish manually [172]. These examples suggest that AI-assisted nanosensing may be especially valuable for multiplex detection, adulteration screening, spoilage classification, and pattern-based contamination surveillance.

However, digital and AI-assisted food safety systems require careful validation. Smartphone-based sensors are affected by lighting conditions, camera quality, image angle, distance, background color, and user handling. AI models are affected by dataset size, matrix diversity, preprocessing methods, and model bias. A model trained on one food matrix may not perform well in another. For example, a colorimetric model calibrated for shrimp spoilage may not accurately predict spoilage in chicken or pork because the matrix composition, pigment background, and volatile profile are different [173]. Similarly, a SERS model trained on one pesticide and one substrate may fail when exposed to mixed residues, complex extracts, or different fruit surfaces. Therefore, AI-assisted nanosensors should include external validation, independent test sets, matrix-matched calibration, uncertainty reporting, and transparent decision thresholds.

In future food safety systems, nano-enabled digital tools should be integrated with established monitoring programs rather than used as isolated devices. For example, a processing plant could use nanosensor strips for rapid pathogen screening in wash water, IoT temperature loggers for cold-chain verification, smart packaging indicators for retail freshness monitoring, and confirmatory laboratory methods for regulatory decisions. AI could then combine these data streams to identify high-risk lots, recommend corrective actions, and improve recall precision. Such integration would make rapid detection more actionable and could reduce both foodborne illness risk and food waste.

### 6.3. Validation, Standardization, and Regulatory Translation

The translation of nano-enabled sensors into food safety practice depends strongly on validation and standardization. Many published studies report excellent sensitivity, short response times, and low detection limits, but these results are often obtained under simplified laboratory conditions. Recent reviews have similarly emphasized that successful translation of nano-enabled food sensors requires not only high analytical performance but also rigorous validation in complex food matrices, standardized testing protocols, reproducibility, and practical applicability under real-world conditions [174]. A sensor that performs well in buffer may show reduced reliability in milk, meat, seafood, fruit juice, grains, spices, or leafy greens because real food matrices contain proteins, fats, pigments, salts, carbohydrates, enzymes, and background microbiota. Therefore, practical validation must include matrix effects, recovery studies, reproducibility, stability, interference testing, and comparison with reference methods [175].

Several published examples illustrate the importance of matrix validation. Lateral flow assays for mycotoxins are often tested in cereals, nuts, milk, or feed extracts because these products contain matrix components that can suppress or distort immunoassay responses. Electrochemical sensors for heavy metals must account for pH, ionic strength, organic matter, and competing ions [176]. SERS platforms for pesticide residues must be tested on real fruit or vegetable surfaces because wax layers, pigments, and uneven surfaces can influence analyte extraction and spectral intensity [177]. Pathogen biosensors must also address enrichment, viability, and sample size because foodborne pathogens may be present at very low levels and unevenly distributed. Recent reviews on nanoparticle-based pathogen detection emphasize that food matrix complexity and low pathogen abundance remain major obstacles for real-world deployment [178].

Standardization is also needed because nano-enabled sensor studies often report performance using different units, detection conditions, sample preparation steps, and statistical approaches. For example, one pesticide sensor may report detection limits in buffer, another in fruit extract, and another on spiked vegetable surfaces, making direct comparison difficult. Similarly, pathogen sensors may report results as CFU/mL, CFU/g, genomic copies, optical density, or fluorescence intensity [179]. To improve comparability, researchers should report the sensor architecture, nanomaterial synthesis method, particle size distribution, surface charge, bioreceptor immobilization chemistry, number of replicates, matrix type, extraction procedure, recovery rate, response time, storage stability, limit of detection, limit of quantification, and comparison with an accepted method.

**Figure 6:**
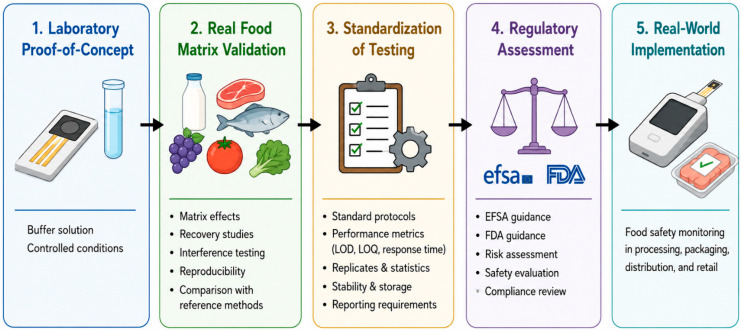
Schematic overview of the validation pathway required for translating nano-enabled food safety sensors from laboratory proof-of-concept to practical implementation (modified by Figure Labs Starter Plan).

Regulatory translation of nano-enabled food safety technologies requires a substantially higher level of evidence than proof-of-concept demonstrations. Recognizing the unique characteristics of nanoscale materials, regulatory agencies have developed specific frameworks to support their safety assessment and oversight. The European Food Safety Authority (EFSA) has issued guidance for the risk assessment of nanomaterials in the food and feed chain, as well as technical guidance for determining the presence of small particles, including nanoparticles, in regulated products [180]. These documents emphasize the importance of characterizing nanoscale attributes such as particle size and size distribution, surface chemistry, dissolution behavior, agglomeration state, and potential exposure pathways when evaluating safety. Similarly, the U.S. Food and Drug Administration (FDA) has published guidance addressing the application of nanotechnology in FDA-regulated products, highlighting that materials exhibiting nanoscale dimensions or nanoscale-dependent properties may require additional consideration during safety evaluation and regulatory review [181]. The overall pathway from laboratory validation to regulatory translation and practical deployment is summarized in Figure 6.

The intended use of the sensor should determine the level of validation required. A sensor used for internal screening in a processing facility may not need the same regulatory pathway as a device used for official compliance testing. A freshness indicator on packaging may require migration and consumer-interpretation studies, while a pathogen biosensor used for release testing may require rigorous comparison with validated microbiological methods. Therefore, authors should define whether their sensor is intended for preliminary screening, quality monitoring, smart packaging indication, environmental monitoring, regulatory testing, or confirmatory analysis. This distinction will prevent overclaiming and help align validation with practical requirements.

Another important issue is interlaboratory reproducibility. Many nanosensors are developed and tested in a single laboratory, but commercial or regulatory adoption requires performance across different users, instruments, batches, and environmental conditions [182]. Future research should include collaborative validation studies, standard reference materials, blind samples, naturally contaminated samples, and field trials. For smart packaging, validation should also include shelf-life studies, temperature fluctuation, package leakage simulation, and consumer readability. For AI-assisted nanosensors, validation should include independent datasets and performance testing across multiple devices and food matrices. Without these steps, nano-enabled detection will remain promising but difficult to translate.

### 6.4. Sustainability, Life-Cycle Thinking, and Implementation Challenges

Sustainability should be treated as a measurable design requirement for nano-enabled food safety systems. Rapid nanosensors can support sustainability by enabling earlier contamination detection, reducing unnecessary recalls, preventing large-scale food waste, lowering sample transport requirements, and decreasing dependence on solvent-intensive laboratory analysis. However, the sensors themselves can create new environmental burdens if they rely on toxic precursors, rare metals, non-biodegradable plastics, single-use components, or poorly controlled nanoparticle release. Therefore, sustainable nano-enabled detection requires life-cycle thinking from material selection to disposal [122,183].

Life-cycle assessment (LCA) is especially relevant for smart packaging and disposable sensors. For example, a single-use nanosensor strip may reduce analysis time and food waste, but large-scale use could generate additional plastic and electronic waste [184]. A smart packaging film may extend shelf life and prevent spoilage, but its environmental benefit depends on the type of polymer, nanomaterial loading, production energy, recyclability, and end-of-life pathway. Nizam et al. reviewed life-cycle assessment studies of nanomaterials and showed that environmental impacts vary strongly depending on raw materials, synthesis processes, energy demand, functional benefits, and disposal assumptions [185]. This finding is important for food applications because the sustainability advantage of nanotechnology should not be assumed solely based on improved performance.

Recent advances in intelligent and active food packaging have increasingly focused on the use of bio-based and biodegradable materials to mitigate the environmental impacts associated with conventional petroleum-derived packaging systems. Biopolymers such as nanocellulose, chitosan, starch, pectin, gelatin, alginate, and polylactic acid have been widely investigated as functional matrices for incorporating sensing and active components into sustainable packaging platforms. Among these approaches, chitosan–anthocyanin composite films have attracted considerable attention because they combine biodegradable polymer matrices with naturally derived colorimetric indicators for real-time freshness monitoring [186]. Similarly, biodegradable films incorporating carbon dots have demonstrated fluorescence-based sensing capabilities while offering a potentially lower-risk alternative to heavy-metal-containing quantum dots [187]. Nanocellulose has also emerged as a promising reinforcement material due to its ability to enhance mechanical strength, improve barrier performance, and reduce dependence on petroleum-based plastics in next-generation packaging systems [188]. These examples show how sustainable material design can be aligned with sensor function. However, biodegradability alone is not enough. The final composite may behave differently from the base polymer, especially when metal nanoparticles, crosslinkers, plasticizers, or synthetic dyes are added.

Implementation also depends on cost, scalability, and user acceptance. A sensor may be scientifically advanced but commercially unrealistic if fabrication requires expensive nanomaterials, multi-step synthesis, unstable reagents, or specialized instruments. For food processors, the ideal sensor should be rapid, affordable, easy to use, stable during storage, compatible with routine sampling, and linked to clear corrective actions. For retailers and consumers, the response must be simple enough to interpret without technical training. If a smart label changes color but the meaning is unclear, it may increase confusion or waste of food rather than improve safety. Therefore, future studies should evaluate not only analytical sensitivity but also user readability, false-positive risk, false-negative risk, cost per test, and compatibility with existing food safety workflows.

Nanomaterial safety remains another major implementation challenge. Even when a nanomaterial is immobilized in a film or sensor, migration, abrasion, degradation, and environmental release should be assessed. This is particularly important for silver nanoparticles, metal oxide nanoparticles, graphene-based materials, and persistent nanocomposites. Safe-by-design strategies can reduce risk by selecting lower-toxicity materials, using bio-based supports, immobilizing nanoparticles, reducing loading levels, and designing sensors that are physically separated from direct food contact. An additional approach involves surface functionalization with biocompatible polymers. Hyaluronic acid-coated Cu-ZIF-8 nanocomposites, for example, demonstrated improved colloidal stability and reduced cytotoxicity while preserving excellent antimicrobial activity on food-contact surfaces, illustrating how material engineering can improve safety without compromising functionality [189].

Another illustrative example is the work of Xue et al. (2017), who developed a paper-based SERS sensor in which Au nanoparticles were immobilized on porous polymer nanorods, allowing analytes to be transported by capillary action to the sensing region while the nanoparticles remained fixed within the substrate [190]. This architecture minimized nanoparticle dispersion and enabled highly sensitive detection without direct nanoparticle transfer to the sample, illustrating a practical, safe-by-design approach for food and environmental sensing. In packaging applications, migration testing under relevant food simulants and storage conditions should be included. In disposable devices, end-of-life management should be considered, especially when sensors contain metals or electronic components.

A major research gap is the limited number of studies that combine analytical performance, safety assessment, life-cycle thinking, and real-world testing in one framework. Many papers report excellent detection limits but do not evaluate material fate, toxicity, cost, or scalability. Others focus on biodegradable packaging but provide limited validation against real spoilage or contamination conditions. Future work should integrate these dimensions more systematically. For example, a strong study could compare a carbon dot-based freshness film with a conventional synthetic dye indicator in terms of response accuracy, film stability, migration, cost, biodegradability, and food waste reduction. Similarly, a nanozyme-based pesticide sensor with a data-driven application could be evaluated not only for detection limit but also for reagent toxicity, storage stability, field usability, and waste generation [191].

For sustainable implementation, nano-enabled food safety systems should be designed as part of tiered monitoring programs. Low-cost indicators and portable nanosensors can provide early warnings at farms, processing facilities, transport vehicles, retail displays, and households. Positive or uncertain results can then be confirmed by validated laboratory methods. This approach balances speed with reliability and avoids overdependence on any single technology. The most successful platforms will be those that improve food safety decisions while also reducing waste, minimizing environmental burden, and fitting realistically into food industry operations.

## 7. Conclusions

Sustainable nanotechnology offers a promising pathway for improving rapid food contaminant detection and strengthening future food safety systems. As food supply chains become more complex, conventional laboratory-based methods alone are no longer sufficient to support fast, distributed, and preventive monitoring. Nano-enabled sensors can help address this gap by improving sensitivity, reducing response time, enabling portable detection, and supporting real-time or near-real-time decision-making across production, processing, storage, transportation, retail, and consumer settings.

This review highlights that nanomaterials can improve the detection of a wide range of food contaminants, including foodborne pathogens, mycotoxins, pesticide residues, veterinary drug residues, heavy metals, allergens, adulterants, packaging migrants, and spoilage-related compounds. Metallic nanoparticles, metal oxide nanostructures, carbon-based materials, carbon dots, magnetic nanoparticles, nanozymes, biopolymer-based nanoparticles, and hybrid nanocomposites have been widely incorporated into colorimetric, fluorescent, electrochemical, SERS-based, lateral flow, paper-based, microfluidic, and smartphone-assisted sensing platforms. These technologies provide important analytical advantages, including signal amplification, rapid target recognition, miniaturization, multiplexing capability, and compatibility with portable readout systems.

At the same time, the practical value of nano-enabled detection should not be judged only by low detection limits or laboratory novelty. For food safety applications, sustainable design is equally important. Green synthesis, low-toxicity materials, renewable biopolymers, biodegradable supports, reduced solvent use, safe-by-design strategies, and controlled nanoparticle immobilization are essential for minimizing environmental and health concerns. This is particularly important for food-contact sensors, smart packaging indicators, and disposable testing devices, where migration, exposure, waste generation, and end-of-life management must be carefully considered.

The integration of nanotechnology with smart packaging, Internet of Things platforms, artificial intelligence, machine learning, and digital traceability systems represents a major direction for future food safety monitoring. Such integration can shift contaminant detection from isolated endpoint testing toward connected, predictive, and preventive surveillance. Smart packaging can provide real-time information on freshness, spoilage, gas composition, and storage abuse, while AI-assisted nanosensors can help interpret complex optical, electrochemical, or spectroscopic signals. These systems may improve recall precision, reduce food waste, and support earlier corrective actions before contaminated products reach consumers.

However, several barriers must be addressed before nano-enabled detection can be broadly implemented. Food matrix interference, limited validation in real samples, poor interlaboratory reproducibility, sensor instability, inconsistent reporting standards, and insufficient comparison with reference methods remain major limitations. Many studies still demonstrate proof-of-concept performance in buffer solutions or simplified extracts, but fewer evaluate naturally contaminated foods, commercial processing conditions, long-term storage, or field deployment. Regulatory acceptance also requires clear evidence of nanomaterial safety, migration behavior, exposure potential, and intended use.

Future research should therefore prioritize validated, scalable, and environmentally responsible sensing platforms. Stronger studies should combine analytical performance with sustainability metrics, including material source, synthesis chemistry, toxicity, stability, reusability, migration risk, disposal pathway, and life-cycle impact. Researchers should also clearly define whether technology is intended for preliminary screening, quality monitoring, smart packaging indication, process control, or confirmatory testing, because each use case requires different validation standards. Greater collaboration among food scientists, nanomaterial engineers, toxicologists, regulatory agencies, industry users, and data scientists will be necessary to move these technologies from laboratory prototypes to practical food safety tools.

In summary, sustainable nanotechnology has strong potential to support the next generation of food contaminant detection by making monitoring faster, more sensitive, more portable, and more connected. Its greatest contribution will come from systems that combine reliable sensing performance with safe material design, realistic validation, digital integration, and measurable sustainability benefits. When developed responsibly, nano-enabled detection can contribute to safer foods, reduce waste, improve transparency, and create more resilient food safety systems.

## Data Availability

No new data were created or analyzed in this study. Data sharing is not applicable to this article.
